# Long-term quality of life after surgery for cerebellopontine angle epidermoid cysts: a cross-sectional study

**DOI:** 10.1016/j.bas.2026.106086

**Published:** 2026-05-04

**Authors:** Anselmi Kovalainen, Justiina Huhtakangas, Harri Sintonen, Martin Lehecka

**Affiliations:** a Department of Neurosurgery, Helsinki University Hospital and University of Helsinki, Helsinki, Uusimaa, Finland; b Department of Neurosurgery, Oulu University Hospital, Oulu, Pohjois-Pohjanmaa, Finland; c Department of Public Health, University of Helsinki, Helsinki, Uusimaa, Finland

**Keywords:** Cerebellopontine angle, Epidermoid cyst, Quality of life, Skull base, Patient-reported outcome measures

## Abstract

**Introduction:**

Cerebellopontine angle epidermoid cysts (CPA EC) are rare, benign tumors, which envelop neurovascular structures during their slow growth. Cranial nerve deficits are common in pre- and postoperative phases, and repeated surgery is often needed due to recurrences.

**Research question:**

What is the effect of CPA EC and its surgery on HRQoL?

**Materials and methods:**

We conducted a cross-sectional study of consecutive patients surgically treated for CPA epidermoid cysts at a single center between 1975 and 2022. HRQoL was assessed using a self-administered survey consisting of the 15D instrument, alongside clinical and demographic data. Results were compared to an age-, sex-, and region-matched general population sample.

**Results:**

Of eligible patients, 18/19 (95 %) responded. Median follow-up was 17 years (range 2–48 years). Most patients (78 %) reported persistent symptoms related to CPA EC surgery, notably hearing loss (50 %) and balance issues (50 %). The mean 15D score in the patient cohort was lower than in the general population (difference = 0.037; p = 0.28) representing a clinically important, though statistically non-significant, difference. Usual daily activities (difference = 0.13; p = 0.054) and hearing (difference = 0.084; p = 0.080) were most decreased. Functional independence remained high, with all patients ambulatory (mRS ≤3), and 50 % reported full function (mRS 0).

**Discussion and conclusion:**

Patients surgically treated for CPA epidermoid cysts have a long-term reduction in HRQoL compared to the general population, particularly in domains of day-to-day activities and hearing. While most patients report persistent symptoms, half consider having a normal functional status.

## Highlights

• Patients operated for CPA ECs have a long-term reduction in HRQoL
• Most affected domains of HRQoL are day-to-day activities and hearing
• For comparison of HRQoL, a general population sample was used in the study
• 78 % reported persistent symptoms related to CPA EC and surgery thereof
• Half of patients consider having a normal functional status

## 1 Introduction

Intracranial epidermoid cysts (ECs) are rare benign lesions, two thirds of which are located in the cerebellopontine angle (CPA) (Samii et al., 1996; Yasargil et al., 1989; Mahoney, 1936; Kurucz et al., 2023). Common presenting symptoms are associated with the vestibulocochlear or trigeminal nerve, related to the envelopment of cranial nerves during tumor growth (Kurucz et al., 2023; Berger et al., 1985; Sakamoto et al., 2021; Sellier et al., 2022; Kondziolka et al., 1989; Kovalainen et al., 2025). Surgery is the main treatment option, usually reserved for symptomatic lesions. Cranial nerve deficits are common either due to persistent pre- or postoperative symptoms, or recurrent tumor growth. Recurrences are frequent irrespective of the extent of resection (Shear et al., 2020), and can occur even decades after previous operation (Cuoco et al., 2019). Several reoperations may be required for CPA ECs (Kovalainen et al., 2025).

Due to the benign and congenital nature of CPA ECs (Mahoney, 1936; Kondziolka et al., 1989), evaluation of patients’ postoperative quality of life is of particular importance. While an effect on quality of life is expected based on previous retrospective studies on functional status and evolution of symptoms (Sellier et al., 2022; Shear et al., 2020; Kobata et al., 2002; Schiefer et al., 2008; Czernicki et al., 2016), health-related quality of life (HRQoL) has not been assessed in any of the previous studies. The clinical outcomes are often reported as satisfactory; however, long-term patient-reported outcomes remain underexplored.

To study the patient-reported outcome after CPA EC surgery, we conducted a cross-sectional study focused on HRQoL. The aim of our study is to determine the long-term symptoms and HRQoL as perceived by the patients. We hypothesize that there is a decline in HRQoL, especially in domains most affected by common CPA EC-related symptoms.

## 2 Methods

**Fig. 1:**
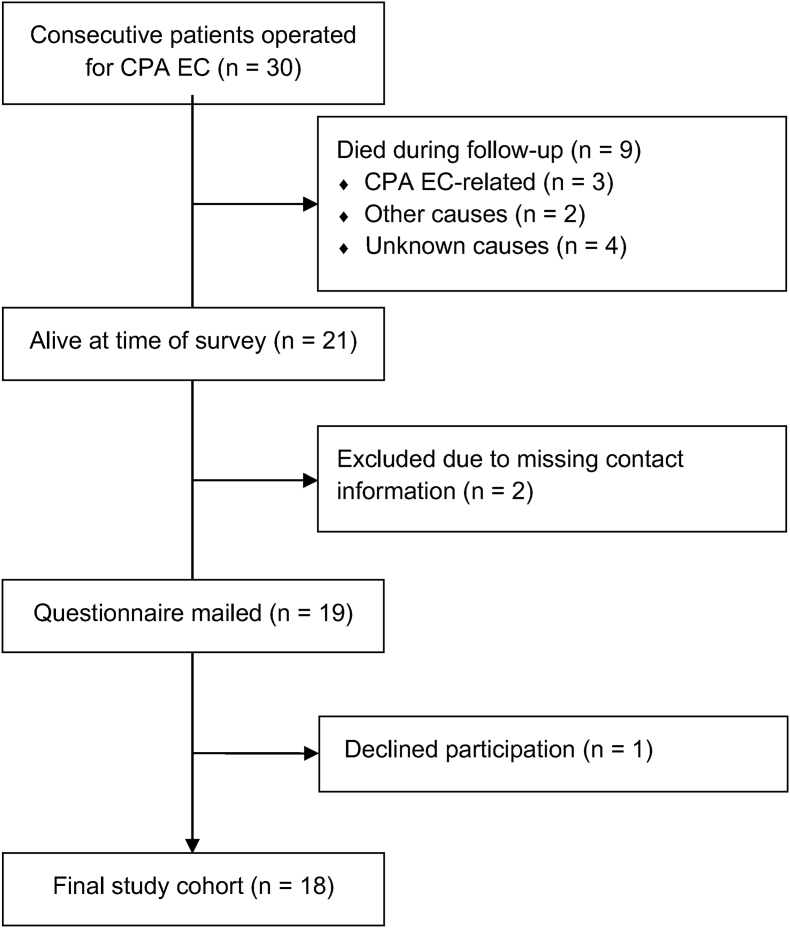
Patient selection process outlined in a flow chart.

This is a cross-sectional survey study of a consecutive cohort of patients operated for CPA EC between 1975 and 2022 at a single academic neurosurgery center with a catchment area of 2.2 million (2023). The diagnosis of CPA EC was confirmed histopathologically in all cases. Only cases with primary tumor volume in the cerebellopontine angle were included. Cases which extended into temporal bone structures were excluded. Clinical data from our previously published retrospective cohort were available, including preoperative symptoms, tumor characteristics, recurrence and reoperations (Kovalainen et al., 2025). Our base cohort consisted of 30 patients, of which 9 had died during follow-up, two patients were excluded due to missing contact information, and one patient did not want to participate in the study. This resulted in the final study cohort of 18 patients. The patient selection process is illustrated in Fig. 1. Of the 9 patients excluded from this study due to death during follow-up, 3 died due to causes related to CPA EC, 2 due to other causes, and 4 for unknown causes, as reported in our retrospective study. As a comparison cohort we used an age-, sex-, and geographical area-adjusted, representative population sample (n = 1265) from a national health survey (Koskinen and Annamari, 2012).

The structured questionnaire used in the study consisted of clinical data, information on smoking, comorbidities, epileptic seizures, Modified Rankin Scale (mRS), and the 15D questionnaire. In the section on persistent symptoms, patients were asked to report symptoms persisting after CPA EC surgery. Written informed consent was obtained for all patients included in this study.

We used the 15D instrument to assess the HRQoL (Sintonen, 2001). It contains 15 different dimensions on the quality of life: mobility, vision, hearing, breathing, sleeping, eating, speech, excretion, usual activities, mental function, discomfort and symptoms, depression, distress, vitality and sexual activity. Each dimension contains five ordinal levels from 1 (best level) to 5 (worst level). The patient was asked to choose one of these levels based on their current condition. The single index score (15D score), representing the overall HRQoL on a 0-1 scale (1 = full health, 0 = being dead) and the dimension level values, reflecting the goodness of the levels relative to no problems on the dimension (=1) and to being dead (=0), were calculated by using a set of population-based preference or utility weights. The minimum clinically important change or difference in the 15D score has been estimated to be ±0.015 (Alanne et al., 2015). The 15D instrument is further described at http://15d-instrument.net/15d/.

Mean differences of 15D scores and dimensions between the patient cohort and population sample were analyzed with independent-samples t-tests. Levene's test was used to determine equal variance, and in case of unequal variance Welch's *t*-test was used. In univariate analyses within the patient cohort, two-group differences were analyzed with independent-samples *t*-test for 15D score, and with Mann-Whitney *U* test for mRS. Kendall's tau-b test was used for associations involving ordinal variables, and Spearman's rho correlation for associations between continuous variables. A p-value of <0.05 was considered statistically significant. No data imputation was used. Statistical analyses were made using IBM SPSS Statistics 29.0.2.0 software.

The study plan and questionnaire were approved by local ethical and research committees. The study was conducted in accordance with the Declaration of Helsinki. The study follows the STROBE guideline for cross-sectional studies.

## 3 Results

### 3.1 Patient demographics

Of the 21 patients alive during the time of survey, 19 had available contact information and 18 participated to form the final study cohort, which corresponds to a response rate of 95 % among contacted patients and 86 % among living patients. Patient demographics are shown in Table 1. There was slight male predominance (56 %). The median age at first operation was 35 years (range 13–51 years) and 54 years (range 23–79 years) at time of survey. Median follow-up time at survey was 17 years (range 2–48 years), with a total follow-up of 319 patient-years.

**Table 1:** Patient demographics.

Characteristic	n or median	% or range
Sex		
Female	8	44 %
Male	10	56 %
Age at Survey, years	54	23–79
Age at First Operation, years	35	13–51
Follow-up time, years	17	2–48
Smoking	6	33 %
Ex-smoker	4	22 %
**Comorbidities**		
Epilepsy	3	17 %
antiepileptic medication	3	17 %
Seizures in last 2 years	0	0 %
Heart Defect	1	6 %
Hypertension	2	11 %
Diabetes	1	6 %
Alzheimer's disease	1	6 %
Migraine	1	6 %
Bipolar disorder	1	6 %
Endocarditis	1	6 %
Systemic Lupus Erythematosus	1	6 %
Other tumor	2	11 %
Basalioma	1	6 %
Prostate cancer	1	6 %
**mRS**		
0	9	50 %
1	5	28 %
2	2	11 %
3	2	11 %
**mRS decrease related to CPA EC**		
Related	8	44 %
Not Related	1	6 %
Not decreased	9	50 %
**Craniotomies**		
Retrosigmoid	14	78 %
Subtemporal	3	17 %
Frontotemporal	1	6 %
**Extent of Resection**		
*Imaging based EOR, n = 14*		
GTR	2	14 %
NTR	5	36 %
STR	7	50 %
*Surgeon based EOR*		
GTR	5	28 %
NTR	7	39 %
STR	6	33 %
**Reoperations**		
0	12	67 %
1	3	17 %
2	0	0 %
3	3	17 %

mRS = Modified Rankin Scale, EOR = Extent of Resection, GTR = Gross-total Resection, NTR = Near-total Resection, STR = Subtotal Resection.

Six patients (33 %) were active smokers and four (22 %) reported being ex-smokers. Three patients reported both having epilepsy and use of antiepileptic medication, two of them had a seizure as the original presenting symptom of EC.

### 3.2 Patient-reported symptoms and functional independence

Preoperative clinical symptoms and current patient-reported persistent symptoms are shown in Table 2. Fourteen patients (78 %) reported having some persistent symptoms. The most common reported symptoms were hearing loss (50 %) or worsening of balance (50 %). Other gait disturbances and tinnitus were also common (33 %). In total, 72% of patients reported either vestibular or cochlear symptoms, and 28 % reported both. Nine patients (50 %) reported being in full functional capacity (mRS = 0). Of the patients with a lowered functional score (mRS >0), seven (78 %) attributed the decrease mainly to CPA EC surgery and one patient (11 %) reported a combined cause of CPA EC and Alzheimer's disease. All patients were ambulatory (mRS ≤3). All patients were seizure free for the last 2 years (Engel grade I). When asked for history of malignancies, one case of basalioma, and one of prostate cancer were reported.

**Table 2:** Preoperative clinical symptoms and current patient-reported persistent symptoms in the HRQoL cohort.

Persistent Symptom	Preoperative baseline clinical data from retrospective study, n (%)	Current persistent symptoms at survey, n (%)
Hemiparesis	0 (0 %)	3 (17 %)
Vision loss	1 (6 %)	2 (11 %)
Visual field defect	0 (0 %)	1 (6 %)
Diplopia	6 (33 %)	4 (22 %)
Hearing loss	7 (39 %)	9 (50 %)
Tinnitus	1 (6 %)	5 (28 %)
Anosmia	0 (0 %)	1 (6 %)
Facial weakness	2 (11 %)	3 (17 %)
Facial hypoesthesia	4 (22 %)	3 (17 %)
Trigeminal neuralgia	2 (11 %)	0 (0 %)
Dysphagia	0 (0 %)	3 (17 %)
Dysphonia	0 (0 %)	1 (6 %)
Memory loss	0 (0 %)	6 (33 %)
Balance problem	5 (28 %)	9 (50 %)
Vertigo	6 (33 %)	3 (17 %)
Gait disturbance	3 (17 %)	6 (33 %)
Seizures	2 (11 %)	0 (0 %)
Headache	5 (28 %)	2 (11 %)
Any symptom	18 (100 %)	14 (78 %)

N = 18.

Preoperative symptoms were extracted from medical records in the previously published retrospective cohort, whereas current persistent symptoms were self-reported in the HRQoL survey.

### 3.3 Health-related quality of life

**Fig. 2:**
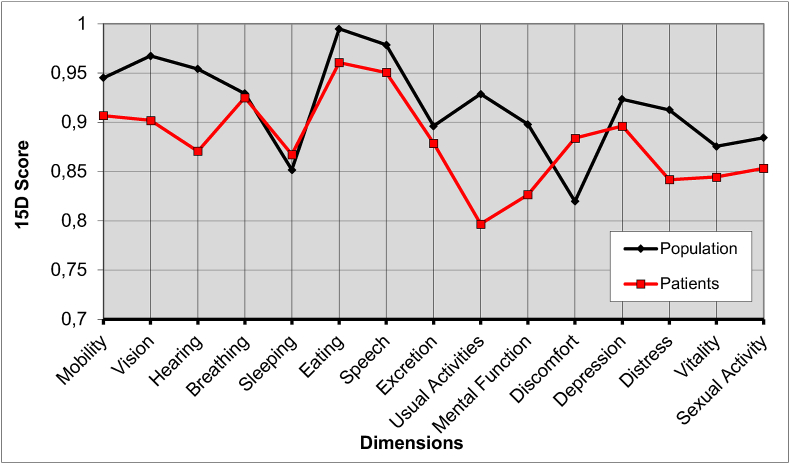
The 15D profile of the patient cohort compared to the general population. No difference of a single dimension score was statistically significant.

**Fig. 3:**
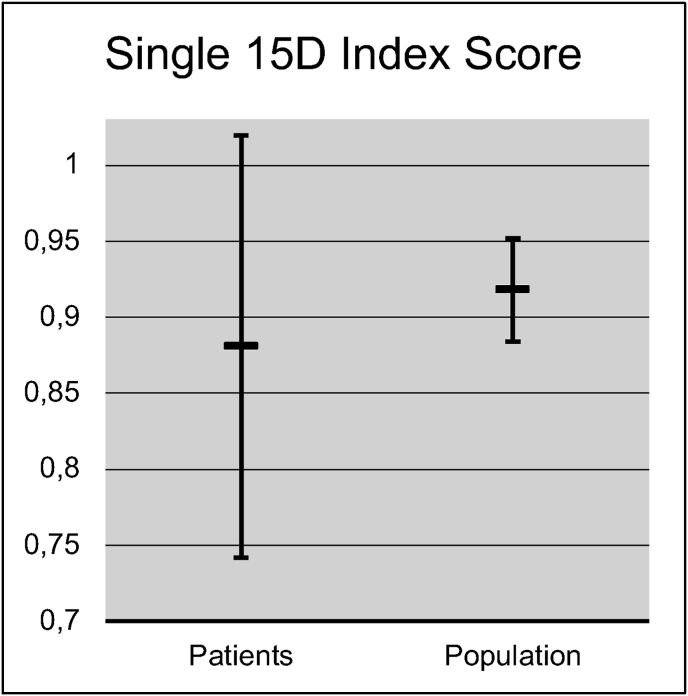
Mean single index 15D score of patient cohort compared to general population. Standard deviation shown with error bar. The differences were not statistically significant, p = 0.283.

Fig. 2 shows the 15D results of the study cohort compared to the general population. While not statistically significant, there was a clinically important difference (Alanne et al., 2015) in the overall 15D score in favor of the population sample (difference 0.037, p = 0.28) (Table 3) (Fig. 3). Of the individual 15D dimensions, the most pronounced decreases compared to the population sample were seen in the “usual activities” (e.g. employment, studying, housework, free-time activities) (difference 0.13, p = 0.054) and “hearing” (difference 0.084, p = 0.080), while a difference in favor of the patient cohort was seen on the dimension of “discomfort and symptoms” (difference 0.0641, p = 0.159).

**Table 3:** The 15D results in the patient cohort and population sample.

	Group	Mean	SD	Difference	p-value
Mobility	Population	0.945	0.062	0.038	0.361
	Patients	0.907	0.163		
Vision	Population	0.967	0.028	0.065	0.139
	Patients	0.902	0.177		
Hearing	Population	0.954	0.044	0.084	0.080
	Patients	0.871	0.187		
Breathing	Population	0.929	0.057	0.004	0.926
	Patients	0.925	0.178		
Sleeping	Population	0.852	0.054	0.016	0.772
	Patients	0.867	0.219		
Eating	Population	0.995	0.008	0.034	0.223
	Patients	0.961	0.114		
Speech	Population	0.979	0.023	0.028	0.319
	Patients	0.951	0.114		
Excretion	Population	0.896	0.062	0.017	0.714
	Patients	0.879	0.188		
Usual Activities	Population	0.929	0.047	0.132	0.054
	Patients	0.797	0.267		
Mental Function	Population	0.898	0.066	0.071	0.182
	Patients	0.827	0.209		
Discomfort	Population	0.820	0.034	0.064	0.159
	Patients	0.884	0.182		
Depression	Population	0.924	0.029	0.027	0.537
	Patients	0.896	0.182		
Distress	Population	0.913	0.047	0.071	0.242
	Patients	0.842	0.244		
Vitality	Population	0.876	0.042	0.031	0.442
	Patients	0.845	0.163		
Sexual Activity	Population	0.884	0.095	0.031	0.648
	Patients	0.853	0.268		
15D Score	Population	0.918	0.034	0.037	0.283
	Patients	0.881	0.139

In univariate analyses, age at primary operation or preoperative tumor diameter did not correlate significantly with the 15D score or mRS score (Table 4). Patients with an infectious event (wound infection, meningitis, aseptic meningitis) (difference 0.25, p = 0.037) experienced a lower 15D score that was both clinically important and statistically significant compared to patients without those events.

**Table 4:** Univariate analyses of CPA EC patient cohort.

Continuous variables	n	Median (range)									Test statistic for 15D score	p-value	Test statistic for mRS score	p-value

Age at primary operation	18	35 years (13–51)									rho = −0.42	0.082^a^	tau = 0.17	0.386^b^
Preoperative tumor diameter	14	33 mm (18–50)									rho = 0.25	0.382^a^	tau = −0.03	0.906^b^

**Categorical variables**	**n**	**Group 1**	**n**	**15D score, mean (SD)**	**mRS, median (range)**	**Group 2**	**n**	**15D score, mean (SD)**	**mRS, median (range)**	**Mean difference in 15D score (95% CI)**		**p-value**		**p-value**

Sex	18	Male	10	0.902 (±0.152)	0 (0–3)	Female	8	0.851 (±0.126)	1 (0–3)	0.050 (−0.092 to 0.192)		0.464^c^		0.095^d^
Smoking at any time	18	No	10	0.883 (±0.127)	1 (0–3)	Yes	8	0.876 (±0.162)	1 (0–3)	0.007 (−0.137 to 0.152)		0.916^c^		0.848 ^d^
Time of primary operation	18	1999 or earlier	7	0.810 (±0.182)	2 (0–3)	After 2000	11	0.915 (±0.105)	0 (0–1)	−0.105 (−0.296 to 0.086)		0.232^c^		0.071 ^d^
Infectious event	15	No	11	0.960 (±0.054)	0 (0–2)	Yes	4	0.713 (±0.142)	2 (0–3)	0.247 (0.028 to 0.466)		**0.037∗**^c^		**0.023∗**^d^
Reoperation due to recurrence	18	No	12	0.923 (±0.106)	0 (0–1)	Yes	6	0.794 (±0.167)	2 (0–3)	0.129 (−0.007 to 0.265)		0.061^c^		0.071 ^d^

∗p < 0.05.

Exact p-values are used due to small sample size.

Statistical tests: a = Spearman's rho, b = Kendall's tau-b, c = independent-samples *t*-test, d = Mann-Whitney U.

Positive mean difference is in favor of group 1.

A difference of ±0.015 in the 15D score is clinically important.

## 4 Discussion

Cerebellopontine angle epidermoid cysts and their surgery are likely to have a negative effect on health-related quality of life. Compared to the general population, the most pronounced differences were seen in patients’ ability to carry out daily activities and hearing-related quality of life. Although 78 % of patients reported persistent symptoms related to CPA EC, their functional independence was generally preserved.

### 4.1 Long-term symptoms

Decades after CPA EC surgery, 78 % of patients report long-lasting symptoms, most commonly hearing loss, and balance or gait disturbances. Similarly, our previous retrospective study from the same base cohort found 16 % to be asymptomatic at end of follow-up (Kovalainen et al., 2025). While persistent symptoms are known to be common after surgery for CPA EC, the rate in our study was higher than in previous retrospective studies by other authors. Schiefer & Link (Schiefer et al., 2008) reported preoperative symptoms to persist in 58 % of patients. Gopalakrishnan et al. (2014) reported 55 % (21/38) and Czernicki et al. (2016) reported 65 % (11/17) of patients to have a reduced functional outcome (mRS >0) at end of follow-up. Sellier et al. (2022) reported improvement in 64 % of preoperative deficits, while persistent symptoms are not quoted. The difference between our findings and previous studies may be explained by documentation bias and underreporting of mild symptoms in retrospective studies based on patient records. Also, often no distinction was made between improvement and complete resolution of a symptom.

In our survey study, we specifically asked patients to report symptoms related to CPA EC and its surgical treatment. We observed an apparent inconsistency between the reported persistent symptoms (78 % report some symptom) and full functional status (50 % report mRS = 0, no symptoms). This likely reflects differences in what these measures capture. When deliberately asked, patients may more often report even mild symptoms, such as hearing loss, tinnitus or balance problems. Conversely, mRS is a global measure of functional status, and patients may primarily report symptoms perceived relevant to their functional capacity when selecting a mRS category. Over time, patients may adapt to their residual limitations and may not perceive them as constantly affecting their everyday life. Thus, patients may report residual symptoms while considering their overall functional status to be normal or near-normal.

### 4.2 Functional outcome and health-related quality of life

The functional outcome after our extensive follow-up was generally favorable; half reported a full functional outcome (mRS = 0), with a further 28 % able to continue full activities despite symptoms (mRS = 1). Additionally, the higher score on the 15D dimension of “discomfort and symptoms” could reflect patients’ satisfaction with their current functional status. Previous literature shows similar functional results; 84-89 % of patients have a good, independent outcome, with a non-ambulatory outcome limited to 0 to 24 % (Sellier et al., 2022; Schiefer et al., 2008; Czernicki et al., 2016; Gopalakrishnan et al., 2014).

Despite generally favorable functional outcomes, the single index 15D score of CPA EC patients was clinically importantly reduced compared to the general population. Of the individual 15D dimensions, patients' ability in everyday activities and hearing were the most affected domains. While statistical significance is difficult to achieve in single-center studies due to the rarity of CPA ECs (Shear et al., 2020), i.e. small sample size, when interpreted along with the functional outcome reported above, a decrease in overall HRQoL seems plausible. Furthermore, the most affected HRQoL dimensions correspond to common symptoms reported in retrospective studies (Sellier et al., 2022; Kovalainen et al., 2025; Czernicki et al., 2016). As would be expected, an infectious event during the clinical course negatively affects patients’ HRQoL.

### 4.3 Dysfunction of vestibulocochlear system

In a median follow-up of 17 years after surgery for CPA EC, half of the patients reported some level of persistent hearing loss. Lower hearing-related HRQoL also separated CPA EC patients from the general population. In retrospective literature, 10-55 % of patients are reported to have hearing loss in the postoperative phase (Sellier et al., 2022; Kobata et al., 2002; Schiefer et al., 2008; Gopalakrishnan et al., 2014; Yawn et al., 2016; Papazian et al., 2025; Miller et al., 2012). In studies on vestibular schwannoma, the most common CPA tumor, both overall and hearing-related HRQoL were decreased compared to general population cohorts (Shaffer et al., 2010; Carlson et al., 2015; Tveiten et al., 2017). Interestingly, in another common CPA tumor, petroclival meningiomas, hearing loss seems to be somewhat less prevalent, from 9 to 35 % reported (Gillard et al., 2023; Di Carlo et al., 2020). Although hearing loss is a more direct risk in vestibular schwannoma surgery (Grauvogel et al., 2010), our results suggest that hearing loss has a meaningful impact on the long-term HRQoL of CPA EC patients as well.

Persistent symptoms of the vestibular system, such as balance problems, vertigo and gait disturbances, are also commonly reported in the present study and in prior literature (Sellier et al., 2022; Kovalainen et al., 2025). Although no 15D dimension measures vestibular problems directly, they presumably contribute to the decrease in HRQoL, similar to vestibular schwannoma and meningiomas (Shaffer et al., 2010; Carlson et al., 2015; Tveiten et al., 2017; Grauvogel et al., 2010).

### 4.4 15D and other instruments for assessment of HRQoL

The 15D instrument is a validated, generic HRQoL survey designed to give an assessment of different health-related facets of quality of life (Sintonen, 2001). Although it has been used previously in other neurosurgical patient groups, no direct comparisons can be made to CPA ECs (Pohjola et al., 2019; Kissling et al., 2024; Karppinen et al., 2016; Salo et al., 2002; Jesse et al., 2022; Mainio et al., 2006). In a study on brain arteriovenous malformations, 15D proved a more sensitive marker than Modified Rankin Scale or EQ-5D for measuring patient differences, specifically in patients with low disability (Pohjola et al., 2021). The 15D instrument seems sensitive for hearing-related HRQoL in our study and previous studies (Niemensivu et al., 2015; Wolff et al., 2024). However, the evaluation of vestibular symptoms is only achieved indirectly through “mobility” and “usual activities” dimensions. The effect of vestibular symptoms on HRQoL may be similarly underestimated in CPA EC like in other CPA tumors (Grauvogel et al., 2010). Consequently, an additional instrument, such as the Dizziness Handicap Inventory (Jacobson et al., 1990; Adegboyega et al., 2022), could be paired with the 15D in future studies to better capture the effect of vestibular symptoms on HRQoL of CPA EC patients.

### 4.5 Limitations

Due to the cross-sectional design of our study, we cannot determine within-patient changes in HRQoL. Although preoperative clinical data were available from our retrospective cohort, preoperative HRQoL data were not. Therefore, changes in HRQoL over time cannot be directly evaluated. Furthermore, it is not possible to distinguish the individual effects of CPA ECs and the surgical intervention, especially after long follow-up. Therefore, our aim is to present their combined effect on patients’ HRQoL and persisting symptoms, and have included a general population cohort for comparison to aid interpretation. Due to the rarity of CPA EC, the published studies are largely based on retrospective cohorts of 30–50 patients, often with a relatively short follow-up of 5 years (Shear et al., 2020). Though our patient cohort is limited in size, it represents a true cross-sectional view with an extended median follow-up of 17 years, with patients operated by different surgeons in different time periods. However, because of the lack of statistical power in the present study, our results should be interpreted with caution. Because some patients had died related to CPA EC, survival bias cannot be excluded. There is a need for a larger, multicenter study to confirm our findings. Additionally, a prospective study design would have the opportunity to include objective assessments for clinical parameters, such as status of hearing, in addition to the HRQoL data.

## 5 Conclusions

Patients who were surgically treated for CPA epidermoid cysts have a long-term reduction in HRQoL compared to the general population, particularly in domains of day-to-day activities and hearing. While most patients report persistent symptoms, many consider having a normal functional status. Patients with CPA EC should be informed on the long-term effects of the disease, particularly in presence of vestibular or hearing-related symptoms.

## Declaration of competing interest

The authors declare that they have no known competing financial interests or personal relationships that could have appeared to influence the work reported in this paper.
